# A serum-free medium formulation efficiently supports isolation and propagation of canine adipose-derived mesenchymal stem/stromal cells

**DOI:** 10.1371/journal.pone.0210250

**Published:** 2019-02-27

**Authors:** Laxminarayana R. Devireddy, Michael Myers, Rudell Screven, Zhuoming Liu, Lynne Boxer

**Affiliations:** 1 Division of Applied Veterinary Research, Center for Veterinary Medicine, US Food and Drug Administration, Laurel, Maryland, United States of America; 2 Office of New Animal Drug Evaluation, Center for Veterinary Medicine, US Food and Drug Administration, Rockville, Maryland, United States of America; Faculty of Animal Sciences and Food Engineering, University of São Paulo, BRAZIL

## Abstract

Medium containing Fetal Bovine Serum (FBS) provides a supportive environment for isolation and expansion of mesenchymal stromal/stem cells (MSCs); however, the inherent variability of FBS may contribute to inconsistencies in cell growth and yield between batches of stem cell products. For this reason, we set out to develop a serum-free medium capable of supporting the *in vitro* expansion of MSCs. First a naïve serum-free medium was formulated by Sato’s approach. Once it was established that the naïve serum-free medium supported the expansion of canine adipose-derived MSCs (Ad-MSCs), the serum-free medium was optimized by addition of growth factors. Combinations of growth factors were chosen and compared by their effect on cell proliferation and colony formation. Growth characteristics of canine adipose-derived MSCs cultured in the serum-free medium were comparable to those cultured in standard FBS containing medium. In addition, cell surface marker expression and differentiation potential of serum-free and FBS-based cultures were also comparable. However, a commercial serum-free medium developed for human MSC culture did not support growth of canine Ad-MSCs. In summary, canine Ad-MSCs isolated and cultured in serum-free medium retained the basic characteristics of MSCs cultured in FBS containing medium.

## Introduction

Cell therapies utilizing stem cells are being explored in veterinary clinical practice. Amongst different stem cells, mesenchymal stem/stromal cells (MSCs) are a favored cell type by clinicians and academics alike partly because of their ease of isolation [1, 2]. MSCs are post-embryonic, self-renewing cells, which are capable of giving rise to a variety of parenchymal cells *in vitro* when stimulated with inducers [3]. MSCs are also clonogenic and form stromal progeny *in vitro*, express general fibroblastic markers and when transplanted, modulate the host immune system [4]. MSCs are transient cells that are believed to have a limited life span in the recipient [5]. Additional potentially beneficial properties include the ability to home to sites of damage and inflammation, and secrete trophic factors that influence the repair of damaged tissues. However, real *in vivo* effects of manufactured human or veterinary MSCs are variable [3, 6, 7].

Although MSCs can be isolated from every postnatal tissue, typically fat tissue or bone marrow are prime sources for MSCs due to their relative ease of isolation. Because their numbers in adult tissues are low, MSCs are typically culture expanded to attain a sufficient quantity [8]. A variety of methods and media exist for *in vitro* cultivation of MSCs. Variations in isolation methods or culture conditions such as culture reagents, culture vessels and culture environment contribute significantly to the heterogeneity of MSCs. A few media are described in the literature for both the isolation and expansion of human or veterinary MSCs from fat tissue and bone marrow. Typically, they range from Minimum Essential Medium (MEM) to Dulbecco’s Modified Eagle Medium (DMEM), which are supplemented with fetal bovine serum (FBS) at 10–20% (v/v). FBS provides attachment factors, growth factors and a host of other nutrients. Concentrations of these factors and nutrients in FBS vary greatly amongst suppliers and can additionally vary amongst batches even when obtained from the same supplier. Thus, utilizing FBS containing uncharacterized components contributes to the heterogeneity of MSC number and quality when switching between lots [9, 10]. While this may not be an issue from an academic stand point, for regulatory purposes consistency in the quality of batches of MSCs is critical in the manufacturing process. Some of the growth factors present in FBS also promote differentiation of stem cells [11]. FBS can also be a source of adventitious pathogens and contains serum proteins that have the potential to elicit immune response in recipients. Safety, efficacy, consistency and reproducibility concerns make the proposition of a medium void of FBS attractive.

To overcome the some of the deficiencies associated with the inclusion of FBS in cultivation of MSCs, use of autologous or allogeneic serum, plasma or platelet lysates are proposed for cultivating human MSCs [12]. Similarly, there are reports on the use of blood products for the cultivation of canine MSCs [13]. However, autologous or allogeneic serum or blood products may not be practical for canine MSC expansion because: large amounts of autologous serum may be required for generation of clinically relevant numbers of MSCs; autologous or allogeneic serum derived from adult donors may not contain sufficient growth factors to support growth of MSCs; and allogeneic serum is a potential source of infectious agents [13]. However, these FBS alternatives have the same potential for inducing variability in cell culture as FBS.

While the concept of serum-free medium predominantly devoid of animal components to eliminate variability associated with FBS in MSC production is not novel for cultivation of human and rodent MSCs, efficiency of MSC growth varies depending on the media formulation [11, 14]. Likewise, utilization of serum-free media developed for isolation and expansion of human or rodent MSCs for the expansion of canine MSCs is often met with mixed results [14, 15]. Thus, inconsistencies in growth promoting potential of serum-free media developed for human MSCs on canine MSCs further suggest that unique nutrients or growth stimulants are needed for the cultivation of canine MSCs.

Here we report the development of a serum-free medium for *in vitro* expansion of MSCs from canine adipose tissue (Ad-MSCs). We find that our serum-free medium efficiently supported both derivation and *in vitro* expansion of canine Ad-MSCs. Additionally, canine Ad-MSCs cultivated in this medium exhibited faster growth rates as measured by a lower population doubling (PD) time and a decreased lag phase as compared to canine Ad-MSCs cultivated in serum-containing medium.

## Materials and methods

### Media

#### Serum-containing medium

Serum-containing medium consisted of DMEM/low glucose (Sigma, St. Louis, MO) dissolved into tissue culture grade water (Lonza, Walkersville, MD), filter sterilized and supplemented with 4 mM Glutamax (Life technologies, Carlsbad, CA), 1 x Antibiotic-Antimycotic (Invitrogen, Carlsbad, CA) and 10% heat inactivated fetal bovine serum from HyClone (Logan, UT) here after DMEM/FBS-H or Sigma (St. Louis, MO) here after DMEM/FBS-S or Dog serum (Equibiotech, Kerrville, TX) here after DMEM/DOG-S.

#### Commercial serum-free medium

A commercial serum-free medium developed for expansion of human MSCs was purchased from RoosterBio (Frederick, MD). The composition of this media is proprietary and was not available to the authors.

#### Formulation of serum-free medium in our laboratory

Typically, cells need nutrients (organic nutrients such as carbohydrates, lipids, amino acids, and vitamins; inorganic nutrients such as salts and trace minerals) and non-nutrient factors (such as hormones and growth factors), which are supplied by FBS, and a conducive environment (oxygen, humidity and temperature). Bearing in mind these requirements, we formulated a synthetic medium with defined components. To satisfy the nutrient requirements, we utilized basal media, which included DMEM (contains limited ingredients at higher concentration) and Ham’s nutrient mixture F-12 (contains a variety of ingredients at lower concentration) [16–18]. An equal powder mix (w/w) of these basal media would yield a medium balancing both concentrations and ranges of ingredients. In fact, DMEM/Ham’s nutrient mixture F-12 is often used as a starting formulation for proprietary media [19].

Powder media such as DMEM or Ham’s F-12 are typically supplemented with FBS to provide nutrients and growth factors that are either lacking or present at limiting amounts. A major component of FBS is albumin, which is usually present at concentrations varying from 20 to 50 mg/mL. Concentrations of other components of FBS, such as growth factors, amino acids, carbohydrates, lipids, and hormones are more variable. In addition, FBS also has many undefined components whose role in cell growth and metabolism is unknown. Because our aim is to replace FBS, we supplemented the powder media mix with the most critical components present in serum. FBS can contain enzymes such as lactate dehydrogenase (LDH), alkaline phosphatase (ALP); prostaglandins, endotoxin, hemoglobin, bilirubin, urea, creatinine, prolactin and other unidentified components. However, these components may not be important for cell culture therefore we did not add them to our serum-free medium. Table 1 details all the components in our serum-free medium.

**Table 1:** Composition of serum-free medium for cultivation of canine Ad-MSCs.

Component	Amount	Final	Source	Quality
Water	q.s. to 1 L		Lonza	cGMP
*Powder medium*				
DMEM/F12	1 packet		Invitrogen	cGMP
*Buffering agents*				
Sodium bicarbonate		20 mM	Sigma	Premium
HEPES		5 mM	Invitrogen	cGMP
*Vitamin*				
Glutamax		1.5 mM	Invitrogen	cGMP
*Lipids*				
Lipid concentrate		0.10%	Invitrogen	cGMP
*Hormones*				
Insulin		4 μM	Sigma	GMP
Hydrocortisone		100 nM	Sigma	Premium
Progesterone		17 nM	Sigma	N/A
*Carrier protein/Osmotic*				
BSA	4 g		Sigma	Premium
*Polyamines*				
Putrescine*		56 μM	Sigma	Elite
*Attachment factor*				
Fetuin	1 g		Sigma	N/A
*Antioxidant*				
Asc-2-P		200 μM	Sigma	N/A
*Transport proteins*				
Holo-transferrin		0.35 μM	Sigma	N/A
*Growth factors*				
bFGF		2 ng/mL	Peprotech	Recombinant
PDGF		2 ng/mL	Peprotech	“
EGF		2 ng/mL	Invitrogen	“
TGF-ß1		1 ng/mL	Peprotech	“

*Can be substituted with spermidine and spermine.

N/A—quality control data not available.

#### Serum entities replaced by chemically “defined” components

Nutrients, Lipids and Amino acids. FBS is a major source of extracellular lipids. They are important constituents of cell membranes and serve as fuel and second messengers [20]. In serum-free cell culture systems, lipids are added in the form of lipid concentrates (Thermo Scientific, Carlsbad, CA). They typically contain a mixture of saturated and unsaturated fatty acids, detergents and lipids. To avoid oxidation of fatty acids in this concentrate, we aliquoted small amounts into tightly capped storage tubes with minimal headspace.

The powdered medium (DMEM/F12 mix) contains all the required amino acids. However, we added glutamine to our medium because it is an essential amino acid for protein and nucleic acid synthesis as well as for energy production [21]. Glutamine is extremely unstable at physiological pH and non-enzymatically degrades to ammonia. GlutaMax (L-alanyl-L-glutamine; Invitrogen, Carlsbad, CA), a stabilized form of L-glutamine, is a substitute for glutamine and is more stable in aqueous solutions. We added GlutaMax to a final concentration of 4 mM.

Growth factors: FBS supports cell growth and proliferation due to an abundance of growth factors. To match the growth promoting effect of FBS on MSCs, we added growth factors Fibroblast Growth Factor basic (bFGF), Platelet Derived Growth Factor (PDGF), Transforming Growth Factor-β1 (TGF-ß1) and Epidermal Growth Factor (EGF) [22–24]. to our serum-free medium. We obtained recombinant growth factors from commercial sources and reconstituted these factors in appropriate liquid buffers, aliquoted and stored as per manufacturer’s recommendations. Addition of bFGF to serum-free medium is known to promote proliferation of human MSCs [24]. Here we tested the effect of bFGF on canine Ad-MSC growth by supplementing our serum-free medium with bFGF (Peprotech, Rocky Hill, NJ) at 10 ng/mL. EGF is also a prototypical growth factor for enhancement of human MSC proliferation and growth [25]. Similarly, we supplemented our medium with EGF (Invitrogen) at 10 ng/mL. PDGF is an important mitogen, but its effects on human MSCs are not crucial. However, PDGF in combination with bFGF and TGF-ß1 is shown to promote human MSC proliferation [23, 26]. Therefore, we supplemented our serum-free medium with PDGF-BB (Peprotech, Rocky Hill, NJ) at 5 ng/mL. TGF-ß1 is also reported to be an important promoter of human MSC proliferation together with bFGF [23]. To determine the importance of TGF-ß1 (Peprotech, Rocky Hill, NJ) in canine Ad-MSCs proliferation, we supplemented our serum-free medium at 1 ng/mL. We tested the effect of these growth factors individually or in combination. We combined these growth factors to cover all possible test modalities. In addition, we also tested the synergy of these growth factors on Ad-MSC growth and viability by evaluating different combinations (see Results section for further details).

Proteins: Albumin and Transport proteins: A major protein in FBS at concentrations ranging from 20 to 50 mg/mL. Albumin accounts for nearly 60% of total serum protein. Its main functions are to stabilize extracellular fluid volume and to transport ligands such as hormones, steroids, fatty acids and vitamins [27]. Typical FBS-supplemented media contains 10% FBS, which translates to 2 to 4 g of albumin. Therefore, we supplemented bovine serum albumin specifically, Cohn fraction V (Sigma, St. Louis, MO), which is a relatively defined component at 2 to 4 mg/mL (w/v) to our serum-free formulation.

Transferrin, which transports iron, a trace mineral, is an indispensable requirement for cells as it is a cofactor for metabolic enzymes and enzymes involved in DNA synthesis. Cells acquire iron from carrier proteins such as transferrin therefore we added bovine holo transferrin (Sigma, St. Louis, MO) as a source for iron.

Adherence factors: FBS also contains adhesive factors that aid in attachment of cells to culture surfaces. These factors are largely unknown. Recently, Fetuin-A, a glycoprotein, was shown to be a major serum cell attachment factor [28]. Therefore, we added Fetuin from bovine serum (Sigma, St. Louis, MO) at 0.025% to 0.1% (w/v) to facilitate attachment of canine Ad-MSCs to culture surfaces.

Hormones: Steroid hormones and Non-steroidal hormones. FBS contains adrenocortical hormones such as hydrocortisone (HCN), which is important for adhesion and growth of human MSCs [29, 30]. Typical concentrations of HCN ranged from 100 nM to 1 μM [30]. In this study, the serum-free medium was supplemented with 50 to 100 nM of HCN (Sigma, St. Louis, MO). Progesterone is another steroidal hormone important for modulation of function of MSCs [31]. We added synthetic progesterone (Sigma, St. Louis, MO) to a final concentration of 18 nM.

Insulin is the most frequently supplemented hormone to serum-free medium because it is important for glucose and amino acid uptake, lipogenesis, intracellular transport and the synthesis of proteins and nucleic acids. Insulin also supports enhanced glycolysis, a hallmark metabolic phenotype associated with the undifferentiated state of MSCs by promoting glucose uptake. Typical concentrations range from 4 to 4.5 μM. In this study, we added 4 μM of bovine insulin (Sigma, St. Louis, MO) to the serum-free medium.

Additional media components: We also added the following components, which were found to have an influence on MSC growth. These components such as buffering agents, antioxidants, and polyamines are either absent or present at miniscule amounts in FBS.

Sodium bicarbonate is the most commonly used buffer system. In addition, it is a metabolic precursor. Therefore, we supplemented our media with 1.72 g/L of sodium bicarbonate (Sigma, St. Louis, MO). Bicarbonate buffers require a gas phase enriched in CO_2_ to maintain pH. We have also added HEPES (Invitrogen), an organic buffer, to serum-free medium at 10 mM concentration to provide an additional buffering capacity to thwart undue pH changes during the time that the cultures are out of the incubator and at normal atmosphere for observation and manipulation.

Ascorbic acid stimulates human MSC proliferation without loss of phenotype and differentiation [32]. L-ascorbate 2-phosphate (Asc-2-P) is the most commonly used media supplement for cultivation of stem cells [33]. We added Asc-2-P (Sigma, St. Louis, MO) to serum-free medium immediately prior to use at 200 μM, as higher concentrations seem deleterious for MSCs [32].

The diamine putrescine is a non-protein nitrogen base and the precursor for polyamines spermidine and spermine. Putrescine is associated with proliferation in several mammalian cell lines and important for maintenance of self-renewal in human embryonic stem (ES) cells [34, 35]. Our medium contained 55 μM of putrescine (Sigma, St. Louis, MO).

Non-media components: There are several other factors that might affect serum-free culture of MSCs. The major component of medium is water. Water quality is more critical when cells are grown in serum-free medium than when the same cells are grown with FBS-supplemented medium because the presence of trace minerals and contaminants can adversely impact the performance of the culture medium. We used premium water from Lonza (Walkersville, MD) in our study. In addition to water, media bottles are also important. We used wide-mouthed glass Schott bottles (Fisher Scientific, Pittsburgh, PA) to store medium.

### Tissue culture ware

All tissue culture vessels regardless of size were purchased from Corning (Rochester, NY). Human MSCs growth rates and yields are dependent on the type of culture vessel. To reduce this variation, we coated tissue culture vessels with bovine gelatin (Sigma, St. Louis, MO; 1:20 dilution of 2% solution). Enough gelatin solution was added to cover the surface of the culture vessels and allowed to coat for 30 minutes at room temperature (RT). Plates were inverted to drain excess gelatin solution and plates were air dried prior to use.

### Experimental design

**Fig 1:**
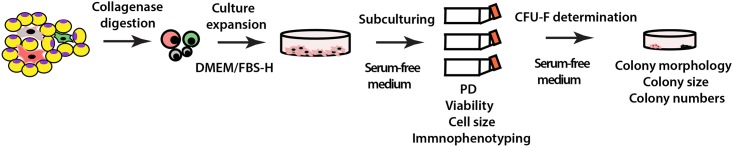
Schematic to evaluate naïve serum-free medium on the expansion of canine Ad-MSCs. Freshly isolated canine Ad-MSCs were cultured in DMEM/FBS-H. Well-developed adherent colonies were recovered by trypsinization and a cell bank was established by cryopreserving a portion of canine Ad-MSCs at passage 1 (P1) for subsequent experiments. Remaining canine Ad-MSCs at P1 were plated in triplicates initially in DMEM/FBS-H, which was exchanged with naïve serum-free medium developed in our laboratory or from commercial serum-free medium. Cell yields, and viability were determined after a 7-day culture period. Canine Ad-MSCs at P1 were also plated in triplicates into culture dishes and CFU-Fs were determined after a 21-day culture period. Colonies were visualized with crystal violet staining and their numbers were enumerated.

The goal of the study was to develop a serum-free medium that supports both isolation and expansion of canine Ad-MSCs. Because previously reported serum-free media were unable to support derivation of human MSCs from tissues, [16, 17] we first derived and cultured canine Ad-MSCs from donors 1–3 in DMEM supplemented with FBS from Hyclone (DMEM/FBS-H) as a starter. Established canine Ad-MSCs were then recultured in the serum-free medium formulated in our laboratory or in a commercial serum-free medium (RoosterBio; see Fig 1 for an experimental strategy). Growth characteristics of canine Ad-MSCs (percent viability, total cell yield, and colony forming unit (CFU) forming capacity) were then compared in our naïve serum-free medium (devoid of any growth factors) to those cultured in commercial serum-free medium or DMEM/FBS-H or DMEM supplemented with FBS purchased from Sigma (DMEM/FBS-S) or DMEM supplemented with allogeneic serum (DMEM/DOG-S). Our intent was not only to compare the growth characteristics of canine Ad-MSCs in serum-free medium to those cultured in serum-supplemented medium but also to compare the growth characteristics of canine Ad-MSCs in DMEM supplemented with various batches of FBS or allogeneic serum.

In a complementary set of experiments, we studied the growth characteristics of canine Ad-MSCs in naïve serum-free medium supplemented with growth factors individually or in conjunction with other growth factors such that they cover all possible combinations. These serum-free media were assessed for total cell yield, percent viability, CFU forming capacity, and CD44 and CD90 expression of canine Ad-MSCs. The serum-free medium that best supported growth of canine Ad-MSCs i.e., naïve serum-free medium supplemented with bFGF and PDGF was further evaluated for its ability to support the primary isolation of canine Ad-MSCs.

### Donor animals

Canine Ad-MSCs were isolated from white adipose tissue, which was purchased from BioreclamationIVT (Westbury, NY). Donor animal details are listed in Table 2.

**Table 2:** Cell yields from processed canine adipose tissue.

Animal number	Breed	Age	Gender	Cell yield/g
Donor 1	Beagle	4.0 y	Female	1.12 x 10^5^
Donor 2	Beagle	3.5 y	Female	1.10 x 10^5^
Donor 3	Beagle	2.3 y	Female	0.68 x 10^5^
Donor 4	Beagle	3.8 y	Female	4.47 x 10^5^
Donor 5	Beagle	3.2 y	Female	0.93 x 10^6^
Donor 6	Beagle	3.0 y	Female	0.68 x 10^6^

Numbers represent total nucleated cells per g of adipose tissue.

### Cells and cell culture

#### Isolation of MSCs from canine adipose tissue

Canine Ad-MSCs were isolated based on adapted methodology [36]. Yields of Ad-MSCs from 6 donors are listed in Table 2. Adipose tissue (~50g each) was thoroughly rinsed with PBS pre-warmed to 37°C (Life technologies, Carlsbad, CA) containing 5% Antibiotic-Antimycotic solution (Life technologies, Carlsbad, CA) until the tissue turned vivid orange or until the opacity of the infranatant did not decline substantially. The tissue was then placed in a sterile culture dish and morseled with a scalpel into an enzymatic solution containing 500 collagenase digestion unit (CDU)/mL of Type 4 collagenase (Worthington, Lakewood, NJ). The tissue and enzyme mixture was subjected to repeat pipetting to further disperse tissue fragments. The enzymatic digestion was continued at 37°C. The reaction was stopped by adding DMEM/FBS-H when <5% of the initial tissue remained. The mixture was then allowed to separate at RT and the non-buoyant fraction was collected into a fresh tube. The separation was further enhanced by adding additional amounts of DMEM/FBS-H pre-warmed to 37°C. Cells were collected by centrifugation at 400 x *g* for 5 min at RT. Collected cells were then washed with PBS containing 2% Antibiotic-Antimycotic solution for a total of 3 washes. Cells were then suspended in red cell lysis buffer (Sigma, St. Louis, MO) and incubated on ice for 10 min to remove contaminating erythrocytes. Cells were separated from lysed erythrocytes by centrifugation at 400 x *g* for 5 min at RT. Cells were then washed twice with PBS containing 2% Antibiotic-Antimycotic solution to remove traces of red cell lysis buffer. Cells were finally suspended into DMEM/FBS-H or serum-free medium and their number was determined in a cell counter. Cells were plated at a density of 30,000 cells/cm^2^ into T-175 flasks and cultured in DMEM/FBS-H or serum-free medium at 0.32 mL/cm^2^. Two days later media was replaced to remove unattached cells. Thereafter, 50% of growth medium was replaced every other day until well-developed colonies appeared. Cells were harvested by trypsinization and resuspended into DMEM/FBS-H or serum-free medium. These cells were designated as passage one (P1) cells.

P1 cells were cryopreserved in DMEM/FBS-H or serum-free medium supplemented with 10% DMSO (Sigma, St. Louis, MO) in liquid nitrogen until further use. To assess cell viability, a vial was thawed rapidly in a 37°C water bath and recovered cells were diluted with 9 mL of DMEM/FBS-H, and centrifuged at 200 x *g* for 10 minutes at RT. Cell pellets were gently suspended in DMEM/FBS-H and cell viability and cell numbers were assessed after staining with Trypan blue in an automated cell counter (Countess, Invitrogen, Carlsbad, CA). All assays described below were performed on cells cryopreserved at P1.

#### Cell proliferation assays

Cells were harvested by trypsinization and viable cells were enumerated in a Cellometer (Life technologies, Carlsbad, CA) following staining with Trypan blue (Sigma, St. Louis, MO). Cells were also observed under Nikon Eclipse Ti microscope and photographed at defined intervals to document the morphology.

#### Colony formation-fibroblast (CFU-F) assays

To determine the clonogenicity of cells expanded in DMEM/FBS-H or serum-free medium with various components, cells were plated onto 100 mm gelatin coated tissue culture dishes. Approximately, 100 cells per dish were plated and fed with the indicated media. A week later medium was refreshed. Colonies were visualized and enumerated following staining with Crystal violet (0.5% solution; Sigma, St. Louis, MO).

Freshly isolated canine Ad-MSCs were plated at varying dilutions onto gelatin-coated 6-well plates and cultured with DMEM/FBS-H or serum-free medium. Cells were incubated in a humidified CO_2_ incubator at 37°C. Medium was replaced 48 hours later to remove non-adherent cells. CFUs were determined after staining with Crystal violet after 14 days of culturing. CFU-F efficiency was determined as the ratio between the number of colonies generated and the number of cells plated.

#### Flow cytometry

To quantitate the composition of canine Ad-MSCs, samples were analyzed by flow cytometry using cell surface markers as described previously [37]. Canine Ad-MSCs were cultured in DMEM/FBS-H or in serum-free medium with or without growth factors for 6 days. Sample cultures were harvested by trypsinization and washed twice with PBS. Single cell suspensions were incubated in a blocking solution containing 5% FBS. Cells were recovered by centrifugation and stained with conjugated antibodies as described previously [37]. After incubation in the dark, cells were washed twice with a wash buffer (1X PBS supplemented with 1% FBS and 0.1% sodium azide), resuspended in blocking solution (wash buffer) and subjected to flow cytometry as described previously [37]. Cell size and granularity were measured using forward- and side-scatter settings. Data were analyzed with FlowJo software (Ashland, OR).

#### In vitro cell differentiation assays

Trilineage (osteogenic, chondrogenic, and adipogenic) potential was evaluated for P1 populations of canine Ad-MSC from the same source isolated and expanded in DMEM/FBS-H or serum-free medium.

**Osteogenic differentiation:** Canine Ad-MSCs isolated and expanded in DMEM/FBS-H or serum-free medium containing bFGF and PDGF were seeded at 1,000 to 10,000 cells/well in a gelatin coated 12-well plate. A day later, osteogenic induction medium (OM) formulated in our laboratory: in-house OM (DMEM/FBS-H or serum-free medium containing bFGF and PDGF supplemented with 200 nM Dexamethasone, 2 mM β-glycerophosphate, 50 μM L-ascorbic acid 2-phosphate—all from Sigma, St. Louis, MO—and 50 ng/mL IGF-1; Peprotech, Rocky Hill, NJ) or OM from commercial source (Osteogenic bullet kit from Lonza, Walkersville, MD) was added to half of the wells, while the other half of the wells were maintained in naïve medium. Media in both groups of wells were completely replaced every 3 days. Cells were cultured for a total of 14 days prior to assessment of mineralization. Cells were washed once with PBS and fixed with 70% ethanol for 5 min at RT. Residual ethanol was removed by washing cells with water twice. Cells were stained with 2% Alizarin Red S (Sigma, St. Louis, MO) for 3 min at RT. Stained cells were then washed 5 times with water and staining was evaluated by light microscopy.

**Chondrogenic differentiation:** Canine Ad-MSCs isolated and expanded in DMEM/FBS-H or serum-free medium containing bFGF and PDGF were seeded at 25,000 cells/well in a gelatin coated 12-well plate. A day later, growth medium in half the wells was exchanged with chondrogenic-induction medium (Lonza, Walkersville, MD). The induction medium was replaced every 3 days for 14 days. Cells were washed with PBS, fixed with 10% neutral buffered formalin and stained with toluidine blue. Staining was evaluated by light microscopy.

**Adipogenic differentiation:** Canine Ad-MSCs isolated and expanded in DMEM/FBS-H or serum-free medium containing bFGF and PDGF were seeded at 30,000 cells/well in a gelatin coated 12-well plate. A day later, Adipogenic Induction Medium (AIM; Lonza, Walkersville, MD) was added to half of the wells. After 3 days of culturing, AIM was replaced with Adipogenic Maintenance Medium (AMM; Lonza, Walkersville, MD). A total of 3 cycles of induction and maintenance was performed for cells in half of the wells. After 14 days of induction/maintenance, cells were carefully washed with PBS, fixed with 10% neutral buffered formalin for 60 min at RT. Cells were washed once with 70% ethanol prior to staining. The cells were incubated with 2% Oil Red O (Sigma, St. Louis, MO) reagent for 5 min at RTF. Excess stain was removed by washing with 70% ethanol. The cells were counterstained with hematoxylin and visualized under a Nikon T2 microscope.

### Statistical analysis

In this study the sample size for each experiment was 3, unless otherwise noted. Means of three replicative measurements of each donor were calculated. Grand means were compared by one-way analysis of variance (ANOVA) followed by the Tukey honestly significant difference (HSD) test for multiple comparisons. For two-way comparison, a Student’s *t* test was used. We utilized GraphPad software for statistical analysis (LaJolla, CA). *p* values less than 0.05 were considered significant.

## Results

### Naïve serum-free medium supports expansion of canine Ad-MSCs

**Fig 2:**
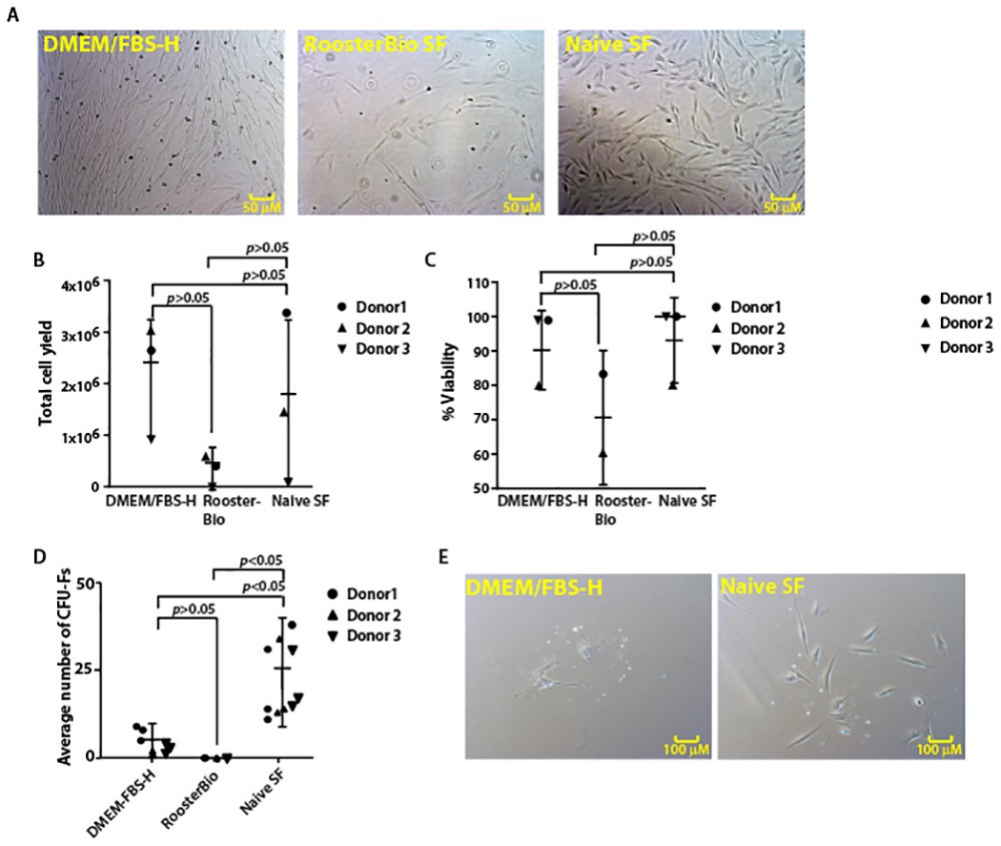
Naïve serum-free medium supports canine Ad-MSC growth. **A**. Images of the cell morphologies of canine Ad-MSCs cultured in indicated medium prior to trypsinization. **B**. Total cell yield of canine Ad-MSCs cultured in indicated medium. Means of two replicates for each donor are shown. **C**. Viability of canine Ad-MSCs cultured in different media. Note: Viability could not be determined for donor 3 in commercial serum-free medium. Means of two replicates for each donor are shown. **D**. CFU-F formation by canine Ad-MSCs in different media. Means of three replicates for each donor are shown. **E**. Colony morphology of canine Ad-MSCs cultured in indicated medium.

We initially tested the ability of serum-free medium formulated in our laboratory on the expansion of canine Ad-MSCs. Canine Ad-MSCs cultivated in naïve serum-free medium displayed growth characteristics like those observed with DMEM/FBS-H (Fig 2A). In contrast, minimal canine Ad-MSC growth was observed when cultured in commercial serum-free medium (Fig 2A). These results suggest that our serum-free medium, even without growth factors, supports expansion of canine Ad-MSCs.

Viability of canine Ad-MSCs and total cell yield were comparable in cells cultured in either DMEM/FBS-H or in naïve serum-free medium (Fig 2B and 2C). In contrast, while not statistically significant, the mean cell viability and mean total cell yield were reduced when canine Ad-MSCs were cultured in commercial serum-free medium (Fig 2B and 2C).

CFU-F potential was higher in cells cultured in naïve serum-free medium compared with CFU-F potential of cells cultured in DMEM/FBS-H or commercial serum-free medium (Fig 2D). Colony size was also higher (>10 cells per colony) in cells cultured in naïve serum-free medium compared with colony size in cells cultured in DMEM/FBS-H (Fig 2E). However, no colonies were formed when canine Ad-MSCs were cultured in commercial serum-free medium. A higher CFU-F potential, together with an increase in colony size, suggests the fraction of canine Ad-MSCs in the total cell population is higher in naïve serum-free medium supplemented cells.

### Delineation of growth factors necessary for canine Ad-MSCs growth

Growth factors such as bFGF, PDGF, EGF, and TGF-ß1 enhance both human and veterinary MSC growth *in vitro* [23, 38–40]. However, it is not clear whether any or all these growth factors are also important for canine Ad-MSC growth. To examine the effect of bFGF, PDGF, EGF, and TGF-ß1 on the growth and expansion of canine Ad-MSCs, we supplemented naïve serum-free medium with these growth factors individually or in combination.

**Fig 3:**
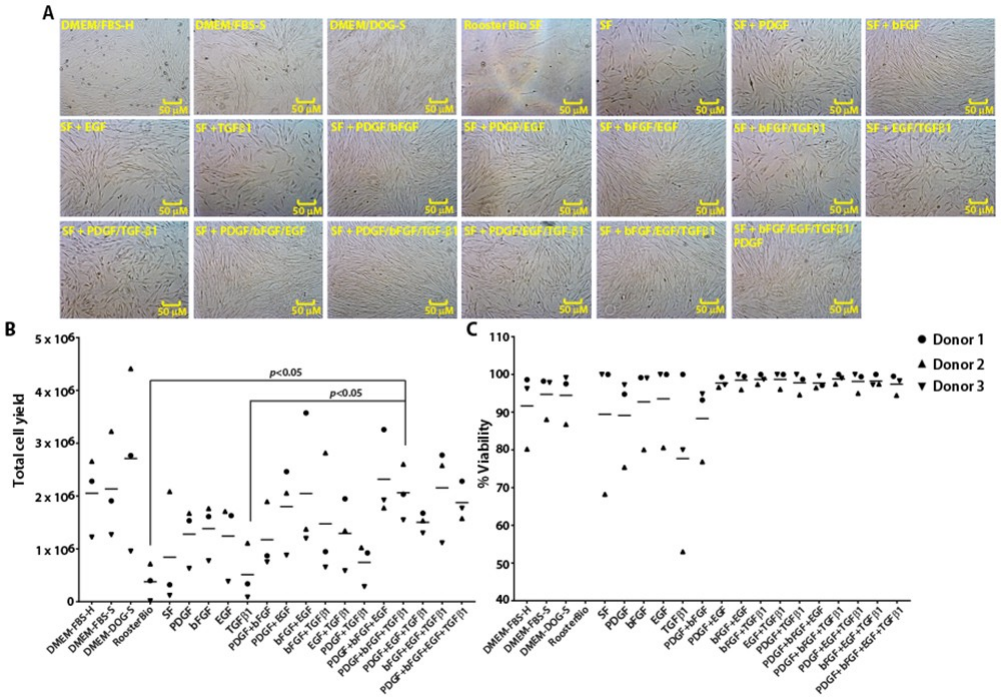
Identification of growth factors required for expansion of canine Ad-MSCs under serum-free conditions. **A**. Photomicrographs of canine Ad-MSCs cultured in serum-free medium with or without growth factors or DMEM/FBS-H or DMEM/FBS-S or DMEM/DOG-S. **B & C**. Cell number and viability were determined a week after placing in serum-free medium containing indicated growth factors or DMEM/FBS-H or DMEM/FBS-S or DMEM/DOG-S. Means of two replicates for each donor are shown.

**Fig 4:**
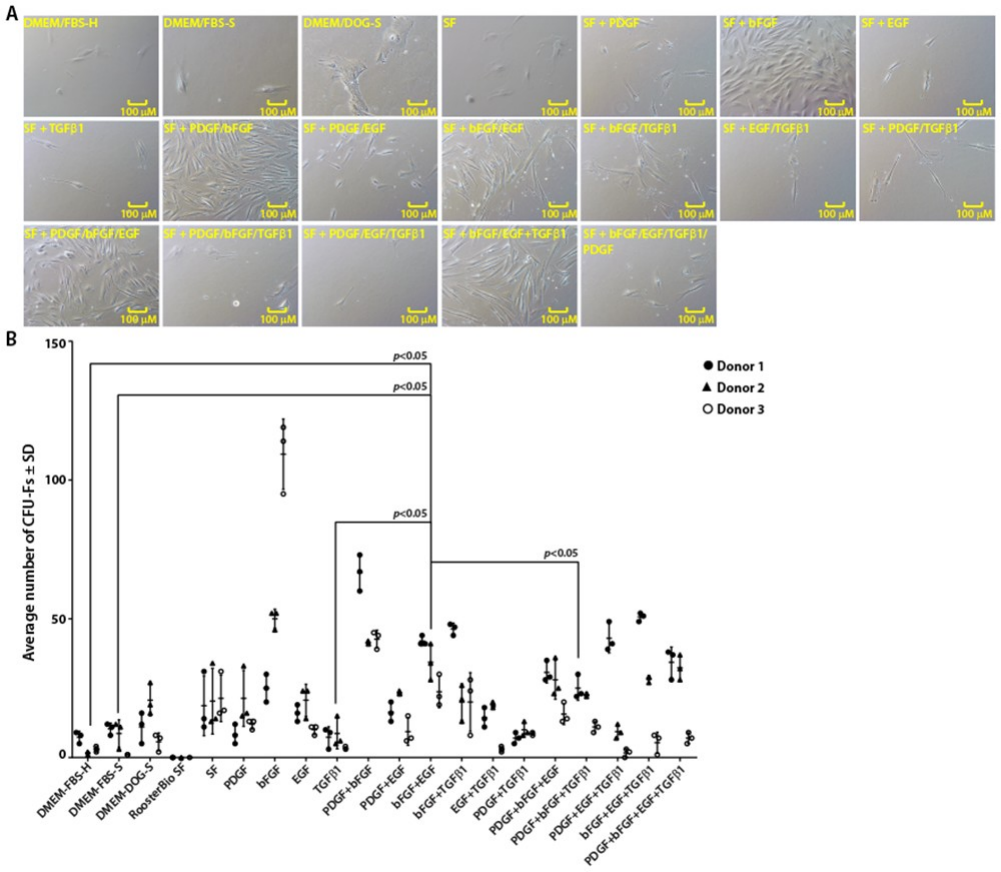
Identification of growth factors required for colony formation by canine Ad-MSCs under serum-free conditions. **A**. Photomicrographs of canine Ad-MSC colonies cultured in serum-free medium with or without growth factors or DMEM/FBS-H or DMEM/FBS-S or DMEM/DOG-S. **B**. Scatter plot of CFU-Fs for canine Ad-MSCs in serum-free medium with or without growth factors or DMEM/FBS-H or DMEM/FBS-S or DMEM/DOG-S. CFU-Fs were enumerated 21 days later. Means of three replicates for each donor are shown.

Data presented in Figs 3 and 4 describe the growth characteristics and CFU-F yields of canine Ad-MSCs cultured in indicated medium, respectively. We found that total cell yields of canine Ad-MSCs cultured in naïve serum-free medium are comparable to serum-based cultures barring donor-to-donor variation (Fig 3). Total cell yields of canine Ad-MSCs from three donors cultured in serum-free medium with or without growth factors are dependent on the type of growth factor as well as the donor (Fig 3). Similarly, cell viability of serum-free cultures is comparable to serum-based cultures except for serum-free medium supplemented with TGF-ß1, which lowered the viability of canine Ad-MSCs in at least two donors (Fig 3B).

We next examined CFU-F ability of canine Ad-MSCs from three donors in both serum-containing or serum-free medium with or without growth factors (Fig 4). Gross evaluation of stained colonies (S1 Fig) and microscopic evaluation of unstained colonies (Fig 4A) suggested that CFU-F ability of canine Ad-MSCs cultured in naïve serum-free medium or serum-containing medium varied depending on the donor. Similarly, the CFU-F ability of canine Ad-MSCs cultured in serum-free medium varied depending on the type of growth factor and the donor. For instance, addition of bFGF to serum-free medium resulted in formation of denser colonies as judged by the intensity of the staining (S1 Fig) and enhanced CFU-F yield in at least two donors (Fig 4B) compared to naïve serum-free medium or serum-containing medium. Addition of PDGF to bFGF-supplemented serum-free medium further increased number of denser colonies and a higher CFU-F yield (S1 Fig and Fig 4B) compared to naïve serum-free medium or serum-containing medium. Supplementation of other growth factors to serum-free medium also affected both colony appearance and CFU-F yield depending on the donor (S1 Fig and Fig 4B).

Addition of TGF-ß1 –a growth factor critical for human MSCs, [41] to serum-free medium resulted in formation of fewer colonies compared to naïve serum-free medium and this effect is donor-dependent (S1 Fig and Fig 4B). This result may partially explain why a commercial serum-free medium developed for human MSCs failed to support growth and clonal expansion of canine Ad-MSCs. However, addition of other growth factors such as bFGF, EGF or PDGF to TGF-ß1-supplemented serum-free medium increased CFU-F yield compared to serum-free medium supplemented with TGF-ß1 or serum-containing medium depending on the donor (S1 Fig and Fig 4B). Since we observed denser and a higher number of colonies in serum-free medium supplemented with bFGF and PDGF compared to naïve serum-free medium or serum-containing medium (S1 Fig and Fig 4B), we decided to perform subsequent experiments in this medium.

### Phenotypic analysis of cell surface markers of canine Ad-MSCs

**Fig 5:**
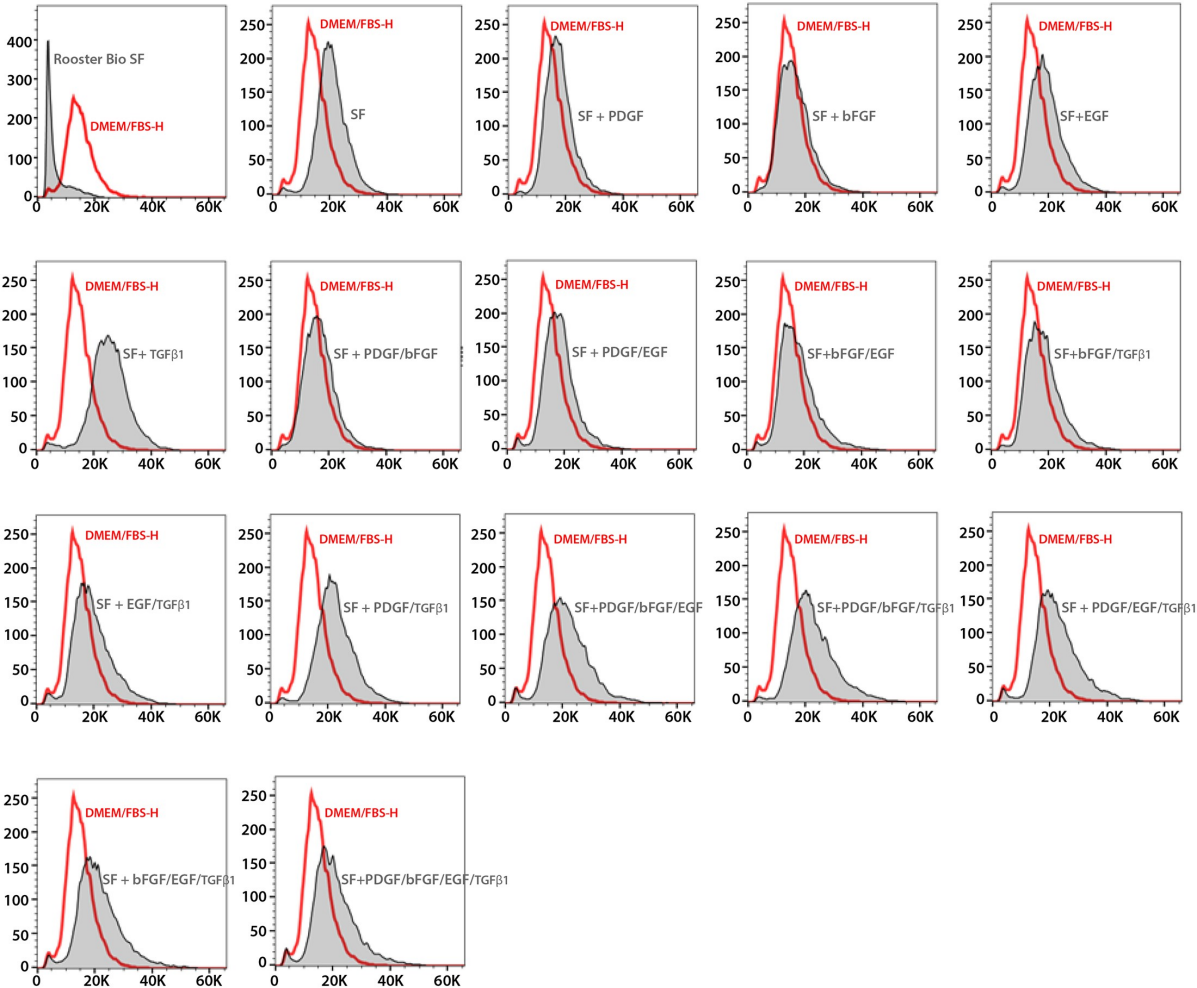
Flow cytometry analysis of canine Ad-MSCs cultivated in DMEM/FBS-H or serum-free medium. Comparison of forward scatters of canine Ad-MSCs cultured in serum-free medium supplemented with the indicated growth factors or DMEM/FBS-H. Y-axis represents mode of the count/number of events and X-axis represents the units of light scatter intensity. SF = naïve serum-free medium.

**Fig 6:**
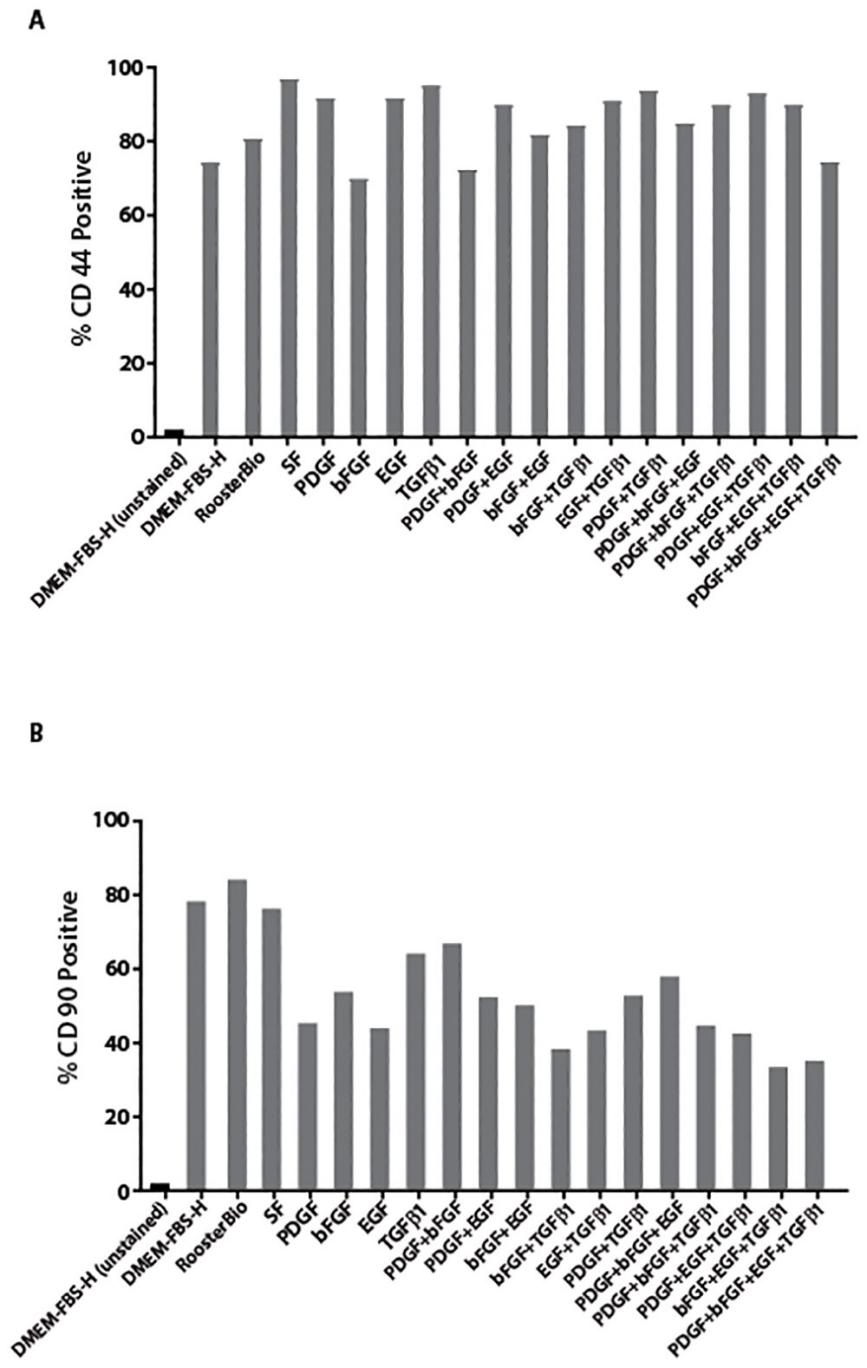
Immunophenotyping of canine Ad-MSCs cultivated in DMEM/FBS-H or serum-free medium. **A**. Analysis for the CD44 expression on canine Ad-MSCs cultured in serum-free medium supplemented with the indicated growth factors or DMEM/FBS-H. **B**. Analysis for the CD90 expression on canine Ad-MSCs cultured in serum-free medium supplemented with the indicated growth factors or DMEM/FBS-H. Y-axis represents % positive cells and X-axis represents the culture medium. SF = naïve serum-free medium.

We previously showed that cultured canine Ad-MSCs express a set of surface markers [37]. To test whether canine Ad-MSCs expanded in serum-free medium also retained the expression of these markers, we performed flow cytometry using specific antibodies. The majority of canine Ad-MSCs cultured in all media types were comprised of small cells (Fig 5). In conformity with previous studies, a majority of cultured canine Ad-MSCs irrespective of culture medium stain positively for both CD44 and CD90 (data from representative samples of each test medium, Fig 6A and 6B) as indicated by the clear shifts in percent positive cells above background. These results suggest that culture media did not impact the cell surface marker expression of canine Ad-MSCs.

### Serum-free medium facilitates isolation of primary canine Ad-MSCs

**Fig 7:**
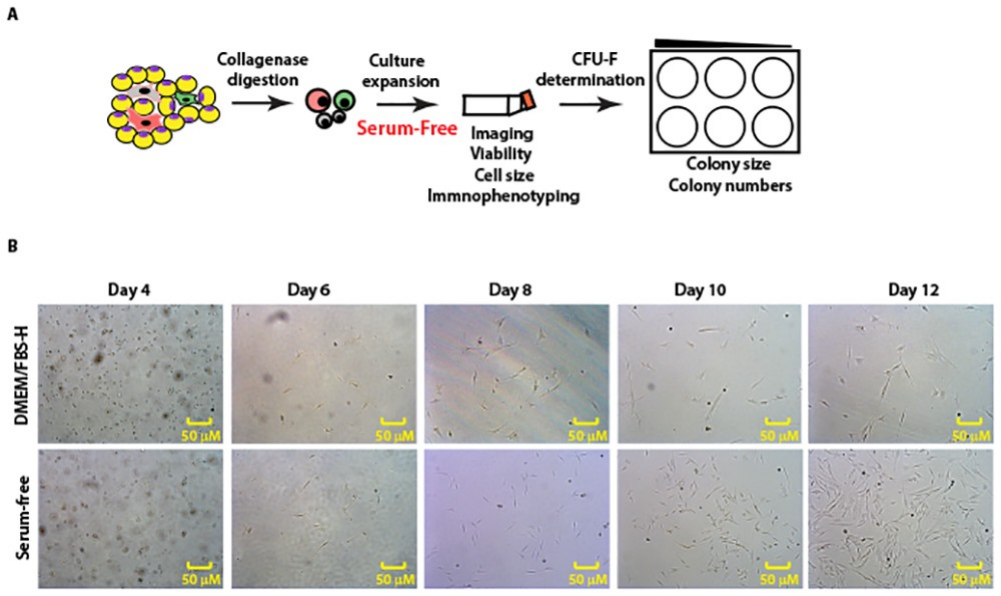
Serum-free medium containing bFGF and PDGF facilitates isolation and expansion of canine Ad-MSCs. **A**. Schematic to evaluate serum-free medium containing bFGF and PDGF for direct derivation and expansion of canine Ad-MSCs. Freshly isolated canine Ad-MSCs from 3 additional donors (donors 4–6) were placed directly into serum-free medium containing bFGF and PDGF. Canine Ad-MSCs were also placed into DMEM/FBS-H to compare the growth characteristics of canine Ad-MSCs in both culture media. **B**. Photomicrographs of canine Ad-MSCs from donor 4 imaged at indicated days of culturing.

Data presented in Figs 3 and 4 suggest that naïve serum-free medium supplemented with bFGF and PDGF is sufficient for the growth, expansion and for colony forming ability of canine Ad-MSCs. We next tested whether this medium can support the direct isolation of canine Ad-MSCs from tissues. Data presented in Fig 7B shows representative images of cells isolated into DMEM/FBS-H or serum-free medium containing bFGF and PDGF at different days post culture. On day 4, we observed sparsely populated cells in both media types. Beginning on day 6, we observed a steady increase in cell number in both media types however the number of cells per microscopic field were higher in the serum-free medium containing bFGF and PDGF. Canine Ad-MSCs in serum-free medium containing bFGF and PDGF were smaller compared to canine Ad-MSCs cultivated in DMEM/FBS-H as judged by microscopy. By day 12, canine Ad-MSCs were nearly confluent in serum-free medium containing bFGF and PDGF whereas canine Ad-MSCs in DMEM/FBS-H were 30 to 40% confluent. These results suggest that both growth rates and cell morphology are different in serum-free medium containing bFGF and PDGF compared to DMEM/FBS-H.

At day 13, canine Ad-MSCs cultivated in serum-free medium containing bFGF and PDGF or DMEM/FBS-H were harvested and their growth rates were compared. Table 3 shows cell yields from the primary culture of canine Ad-MSCs from donors 1–3 in serum-free medium containing bFGF and PDGF and DMEM/FBS-H. Serum-free medium containing bFGF and PDGF consistently supported cell growth in all donors whereas cell growth rates in DMEM/FBS-H were highly variable. In addition, the number of cells obtained from serum-free medium containing bFGF and PDGF cultures is higher compared to DMEM/FBS-H cultures. Cell viability is comparable in both media types. MSCs are estimated to be present at 0.01 to 0.1% in adipose tissue [8] and using this estimate the number of PDs is higher in serum-free medium containing bFGF and PDGF compared to DMEM/FBS-H. Taken together, these results suggest that serum-free medium containing bFGF and PDGF supports efficient growth of canine Ad-MSCs.

**Table 3:** Cell yields comparison of canine Ad-MSC cultured in DMEM/FBS-H or serum-free medium containing bFGF and PDGF.

Animal number	Seeding rate (per cm^2^)	Medium	Culture time (days)	Total cells‡ (10^5^ cells)	PD*	PD/day
Donor 1	30,000	DMEM/FBS-H	13	1.44 ± 0	0.94	0.07
Donor 1	30,000	Serum-free	13	10.69 ± 0.35	4.49	0.34
Donor 2	30,000	DMEM/FBS-H	13	0.053 ± 0	N/A¶	N/A
Donor 2	30,000	Serum-free	13	1.46 ± 0.028	0.96	0.07
Donor 3	30,000	DMEM/FBS-H	13	0.336 ± 0	N/A¶	N/A
Donor 3	30,000	Serum-free	13	4.65 ± 0.15	2.63	0.20

^‡^ Total cell counts were determined from duplicate cultures and the variability represents the range of measured values.

*Number of MSC in adipose tissue is estimated to be 0.1%.

^¶^ A few colonies were observed during the culture period.

### Serum-free medium containing bFGF and PDGF promotes a rapid clonal growth in canine Ad-MSCs

**Fig 8:**
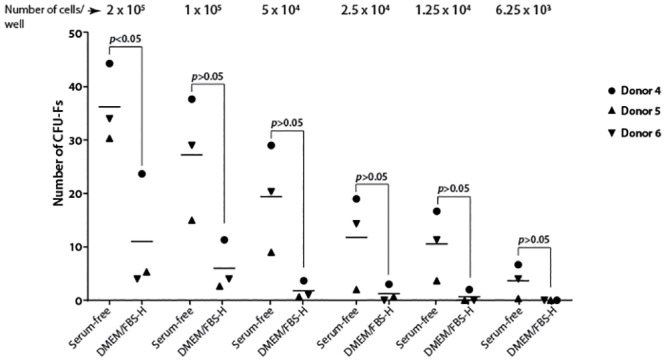
Serum-free medium containing bFGF and PDGF facilitates colony formation by canine Ad-MSCs. Freshly isolated canine Ad-MSCs from 3 additional donors (donors 4–6) were placed directly into serum-free medium containing bFGF and PDGF in parallel with DMEM/FBS-H in serial dilutions indicated on the top of the figure. CFU-Fs were enumerated 21 days later. Means of three replicates for each donor are shown.

We next tested the ability of the serum-free medium containing bFGF and PDGF on the clonal expansion of freshly isolated canine Ad-MSCs. Both the serum-free medium containing bFGF and PDGF and DMEM/FBS-H supported colony formation in canine Ad-MSCs from all three donors. However, canine Ad-MSCs plated in serum-free medium containing bFGF and PDGF formed more colonies than canine Ad-MSCs plated in DMEM/FBS-H only at a lower dilution (2 x 10^5^ cells/well; Fig 8). At higher dilutions, there were no discernable differences in colony formation between SF and DMEM/FBS-H (Fig 8).

### Multipotentiality of canine Ad-MSCs isolated and expanded in serum-free medium containing bFGF and PDGF

**Fig 9:**
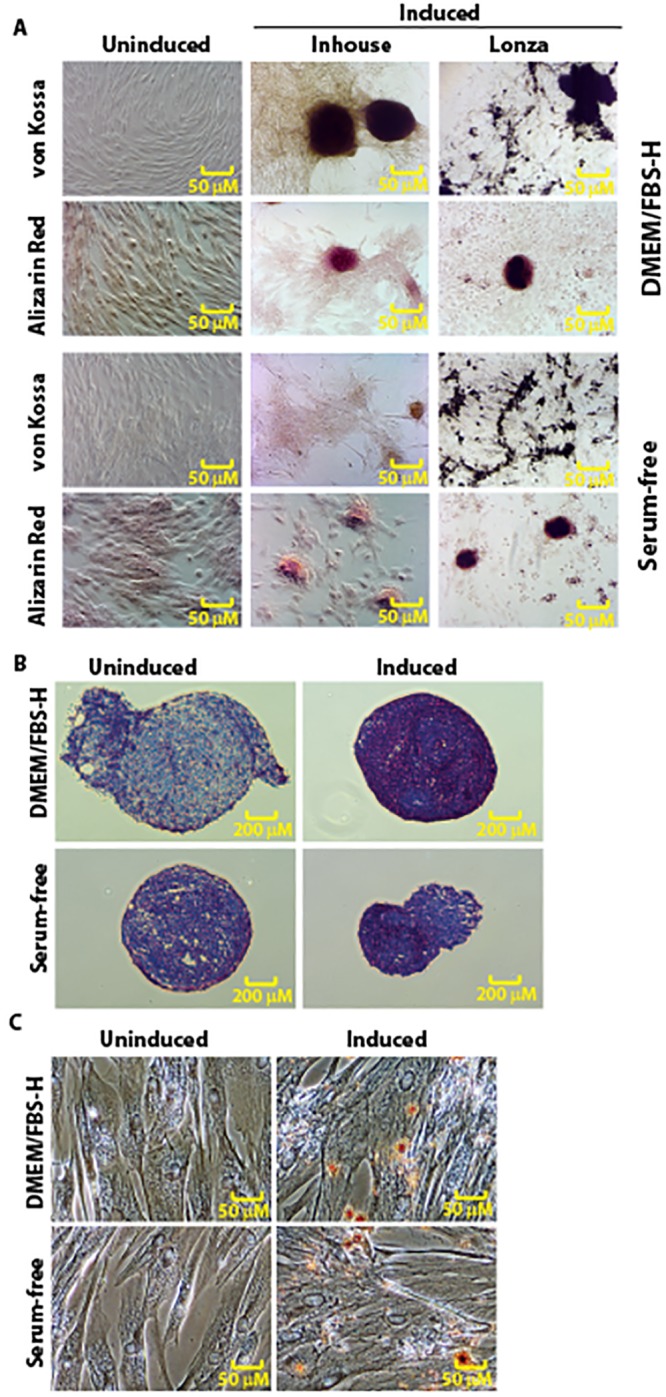
Trilineage differentiation of canine Ad-MSCs expanded in DMEM/FBS-H or serum-free medium containing bFGF and PDGF. **A**. Osteogenic potential of canine Ad-MSCs cultured in DMEM/FBS-H or serum-free medium containing bFGF and PDGF was assessed with Alizarin Red or von Kossa staining after 14 days of induction. Canine Ad-MSCs cultured in both media types formed calcified cell aggregates. **B**. Chondrogenic potential of canine Ad-MSCs cultured in DMEM/FBS-H or serum-free medium containing bFGF and PDGF was assessed with Toluidine Blue staining after 14 days of induction. **C**. Adipogenic potential of canine Ad-MSCs cultured in DMEM/FBS-H or serum-free medium containing bFGF and PDGF was assessed with Oil Red O staining after 14 days of induction/maintenance. Uninduced cells maintained their characteristic spindle shaped morphology. Upon induction, canine Ad-MSCs cultured in both media types formed adipocytes with intracellular lipid droplets.

Canine Ad-MSCs cultured in commercially available osteogenic medium (OM), DMEM/FBS-H or serum-free medium containing bFGF and PDGF supplemented with osteogenic mediators formed an extensive network of dense, multilayered nodules (Fig 9A). We found that induced canine Ad-MSCs were surrounded by a matrix-like substance, presumed to be a collagen I rich extracellular matrix (ECM) as judged by Alizarin Red staining (Fig 9A). To confirm osteogenic differentiation, calcification of the ECM was assessed in induced cells using von Kossa staining (Fig 9A). Calcification appears as black regions (von Kossa) within the cell monolayer. Consistent with osteogenesis, several black regions, indicative of a calcified ECM, were observed in all induced cells. However, the intensity of staining is markedly different in canine Ad-MSCs depending on the type of medium. For instance, the matrix was quite evident in in-house OM treated canine Ad-MSCs compared with canine Ad-MSCs treated with Lonza OM (Fig 9A). In contrast there was no osteogenic differentiation in parallel DMEM/FBS-H or serum-free medium cultures that did not contain osteogenic inducers (Fig 9A). Overall, the staining patterns are not different in induced canine Ad-MSCs cultured in DMEM/FBS-H or serum-free medium containing bFGF and PDGF. These results suggest that the osteogenic ability is retained in canine Ad-MSCs isolated and cultured in serum-free medium containing bFGF and PDGF.

Cells undergoing chondrogenic differentiation are associated with a toluidine blue-positive ECM, indicative of the presence of sulfated proteoglycans within the matrix (Fig 9B). The extracellular matrix staining was similar for canine Ad-MSCs isolated and expanded in serum-free medium containing bFGF and PDGF or DMEM/FBS-H (Fig 9B). These results suggest that canine Ad-MSCs cultured in serum-free medium containing bFGF and PDGF retained the capacity to differentiate toward the chondrogenic lineage.

MSCs cultured in adipogenic induction medium develop lipid droplets, which can be detected using the Oil Red O dye [42]. After exposure to adipogenic induction media, a significant portion of the canine Ad-MSCs cultured in DMEM/FBS-H or serum-free media containing bFGF and PDGF contained multiple, intracellular lipid filled droplets that accumulated Oil Red-O (Fig 9C). The observed cuboidal morphology and lipid accumulation of differentiated canine Ad-MSCs derived in serum-free medium containing bFGF and PDGF were similar to that observed upon treatment of canine Ad-MSCs derived in DMEM/FBS-H (Fig 9C). No lipid droplets were observed in canine Ad-MSC cultures without adipogenic inducers (Fig 9C). These results suggest that canine Ad-MSCs derived in serum-free medium containing bFGF and PDGF undergo adipogenic differentiation.

Combined, all these results suggest that canine Ad-MSCs isolated and expanded in serum-free medium containing bFGF and PDGF retained their plasticity and undergo directed differentiation.

## Discussion

Postnatal stem cells such as MSCs are cultured in a nutrient mixture of salts, sugars, minerals, and vitamins supplemented with biological fluids such as plasma or serum. Because of its widespread availability, serum is most commonly used, especially that sourced from bovine fetuses (FBS). FBS provides factors of critical importance such as nutrients, hormones, growth factors, and carrier proteins. These carrier proteins recognize vitamins, lipids, metals, hormones, attachment and spreading factors, protease inhibitors, and buffering agents whose collective function is to support cell growth. Concentrations of these critical factors vary between batches of FBS, which presents a challenge to standardizing production of an MSC-based product. It is possible that a synthetic medium with defined components may limit variability associated with the use of FBS. Use of serum-free medium to isolate and expand human MSCs from adult tissues is not new [11, 12]. However, the use of serum-free media has not gained prominence for cultivation of veterinary MSCs due to challenges related to cost and availability of defined media that adequately support cells of various species. Serum-free media formulations developed for cultivation of human MSCs have produced mixed results in the culture of canine MSCs [14]. In our study the commercial serum-free medium, that is formulated for human MSCs, did not support cultivation of canine Ad-MSCs suggesting the nutritional and growth requirements of canine MSCs are unique. To date, no serum-free media formulations specific for canine MSCs are reported.

We developed a serum-free medium-based protocol for both the isolation and expansion of canine MSCs from adipose tissue. The comprehensive effects of FBS on cell culture were considered during medium development. These effects include attachment, spreading, growth promotion, and proliferation. Our efforts focused on replacing the growth stimulating activities of FBS with the appropriate combination and concentration of hormones and growth factors necessary to achieve similar cell growth. Using this approach, we formulated a medium, which replaced FBS for both the isolation and cultivation of canine Ad-MSCs. The success of our serum-fee medium also relies, in part, on the use of basal medium (DMEM/F12 mix), which is rich in low molecular weight factors. The use of DMEM/F12 mix is well documented in serum-free media formulations for a variety of cells [19]. Additionally, the components we used in our serum-free medium are defined and of either pharmaceutical or analytical grade.

While the serum-free medium we developed is well-defined, it is not devoid of animal components. The medium contains insulin, bovine albumin, fetuin, and holo-transferrin. Although bovine albumin is a xenogenic protein and presents a risk for immunogenicity reactions in clinical applications, it was chosen because: it is well-characterized, especially the Cohn fraction V; similar consistencies of different batches of Cohn fraction V of bovine albumin; and finally, for its ability to carry fatty acids in a lipid-supplemented media such as our serum-free medium. Canine albumin may be an alternative to bovine albumin if xenogenicity of bovine albumin is a concern. However, unlike bovine albumin, canine albumin is less characterized and Cohn’s process for fractionation of canine albumin is not available and cost prohibitive. Fetuin was included in our medium because it is the principal cell adhesion molecule in FBS [28]. Other available factors that promote cell attachment such as gelatin and fibronectin cannot be added directly to the growth medium. Therefore, we utilized fetuin as a cell attachment agent. Finally, insulin, an important growth factor and facilitator of glucose importer was from bovine pancreas. We chose this source because it is cell culture tested and relatively inexpensive.

Species-specific differences in growth requirements and lack of availability of defined species-specific components present a challenge to the manufacture of veterinary MSCs in a defined medium. This highlights the need for further research on defined species-specific media. The most important aspect of our approach to serum-free culture of canine Ad-MSCs is the derivation of a combination of growth factors for optimal growth. We optimized our naïve serum-free medium with the addition of growth factors required for both the isolation and expansion of canine Ad-MSCs. We found that a set of growth factors (PDGF and bFGF) function synergistically to promote the growth and proliferation of canine Ad-MSCs. In support of these findings, our recent study also demonstrated that both bFGF and PDGF are sufficient for growth, expansion, and multipotentiality of canine Ad-MSCs from three donors [43]. In our study, growth factor requirements for isolation and expansion of canine Ad-MSCs differed from those reported for the growth of human Ad-MSCs. For instance, bFGF and TGF-ß1 promote growth of human Ad-MSC in serum-free medium cultures [41]. However, bFGF supported growth of canine Ad-MSCs, while TGF-ß1 decreased the canine Ad-MSCs population. In addition, the commercial serum-free medium, which was formulated for human Ad-MSCs, failed to support expansion of canine Ad-MSCs. Most commercial serum-free medium contain TGF-ß1, which based on our results, may negatively impact proliferation of canine MSCs.

In this study, although we observed donor to donor variability, the overall growth pattern of canine Ad-MSCs in serum-free medium or DMEM/FBS-H or DMEM/FBS-S is consistent. Other characteristics were also similar for canine Ad-MSCs cultured in serum-free medium or DMEM/FBS-H or DMEM/FBS-S. Trilineage differentiation potential was evaluated as a preliminary assessment of functionality and was similar for canine Ad-MSCs cultured in serum-free medium containing bFGF and PDGF or DMEM/FBS-H. These preliminary results indicate that canine Ad-MSCs isolated and expanded in serum-free medium may retain characteristics similar to canine Ad-MSCs isolated and expanded in DMEM/FBS-H. However, additional research is necessary to assess functionality, further characterize the MSCs, and evaluate additional replicates.

The development of a serum-free medium may be beneficial for both research and clinical applications. Our results indicate that a chemically defined serum-free medium may support the consistent and efficient growth and expansion of canine Ad-MSCs. Due to the potential benefits of using serum-free media, further research is warranted to determine the functionality and *in vivo* potency of these cells. Additional research is also needed to determine the applicability of the serum-free media developed in this study for isolation and expansion of canine MSCs derived from other tissue sources.

## Supporting information

S1 Fig Identification of growth factors required for colony formation by canine Ad-MSCs under serum-free conditions. Images of canine Ad-MSC colonies cultured in serum-free medium with or without growth factors or in serum-containing medium. Small colonies are indicated by arrows. Note: Colonies in TGF-ß1 supplemented serum-free medium are very faint.
(TIF)

## References

[1] Zuk PA. The adipose-derived stem cell: Looking back and looking ahead. Mol Biol Cell. 2010;21: 17831787. 10.1091/mbc.E09-07-0589 PMC2877637 20375149

[2] Zeve D, Tang W, Graf J. Fighting fat with fat: The expanding field of adipose stem cells. Cell Stem Cell. 2009;5: 472–481. 10.1016/j.stem.2009.10.014 19896439 PMC2876189

[3] Bianco P. Mesenchymal stem cells. Annu Rev Cell Dev Biol. 2014;30: 677–704. 10.1146/annurev-cellbio-100913-013132 25150008

[4] Le Blanc K, Mougiakakos D. Multipotent mesenchymal stromal cells and the innate immune system. Nat Rev Immunol. 2012;12: 383–396. 10.1038/nri3209 22531326

[5] Bianco P, Cao X, Frenette PS, Mao JJ, Robey PG, Simmons PJ, et al The meaning, the sense and the significance: translating the science of mesenchymal stem cells into medicine. Nat Med. 2013;19: 35–42. 10.1038/nm.3028 23296015 PMC3998103

[6] Trounson A, McDonald C. Stem cell therapies in clinical trials: progress and challenges. Cell Stem Cell. 2015;17: 11–22. 10.1016/j.stem.2015.06.007 26140604

[7] Arzi B, Mills-Ko E, Verstraete FJ, Kol A, Walker NJ, Badgley MR, et al Therapeutic efficacy of fresh, autologous mesenchymal stem cells for severe refractory feline chronic gingivostomatitis. Stem Cells Transl Med. 2017;6: 1710–1722. 10.1002/sctm.17-0035 28618186 PMC5689767

[8] Baer PC, Geiger H. Adipose-derived mesenchymal stromal/stem cells: Tissue localization, characterization, and heterogeneity. Stem Cells Int. 2012;2012: 812693 10.1155/2012/812693 22577397 PMC3345279

[9] Johnson M. Fetal bovine serum. Mater Methods 2013;3: 175.

[10] Shih DT, Burnouf T. Preparation, quality criteria, and properties of human blood platelet lysate supplements for *ex vivo* stem cell expansion. New Biotechnol. 2015;32: 199–211. 10.1016/j.nbt.2014.06.001 PMC7102808 24929129

[11] Gottipamula S, Muttigi MS, Kolkundkar U, Seetharam RN. Serum-free media for the production of human mesenchymal stromal cells: a review. Cell Prolif. 2013;46: 608–627. 10.1111/cpr.12063 24118248 PMC6496935

[12] Panchalingam KM, Jung S, Rosenberg L, Behie LA. Bioprocessing strategies for the large-scale production of human mesenchymal stem cells: a review. Stem Cell Res Therapy. 2015;6: 225. 10.1186/s13287-015-0228-5 PMC4657237 26597928

[13] Russell KA, Gibson TWG, Chong A, Co G, Koch TG. Canine platelet lysate is inferior to fetal bovine serum for the isolation and propagation of canine adipose tissue- and bone marrow-derived mesenchymal stromal cells. PLoS One. 2015;10: e0136621 10.1371/journal.pone.0136621 26353112 PMC4564274

[14] Clark KC, Kol A, Shahbenderian S, Granick JL, Walker NJ, Borjesson D. Canine and Equine mesenchymal stem cells grown in serum free media have altered immunophenotype. Stem Cell Rev Rep. 2016;12: 245–256. 10.1007/s12015-015-9638-0 PMC4841858 26638159

[15] Schwarz C, Leicht U, Rothe C, Drosse I, Luibl V, Rocken M, et al Effects of different media on proliferation and differentiation capacity of canine, equine and porcine adipose derived stem cells. Res Vet Sci. 2012;93: 457–462. 10.1016/j.rvsc.2011.08.010 21940026

[16] Liu C-H, Wu ML, Hwang SM. Optimization of serum-free medium for cord blood mesenchymal stem cells. Biochem Eng J. 2007;33: 1–9.

[17] Parker A, Shang H, Khurgel M, Katz A.J. Low serum and serum-free culture of multipotential human adipose stem cells. Cytotherapy. 2007;9: 637–646. 10.1080/14653240701508452 17917877

[18] Mather JP. Making informed choices: Medium, serum, and serum-free medium. How to choose the appropriate medium and culture system for the model you wish to create In: Mather JP, Barnes D, editors. Methods in Cell Biol. San Diego, CA: Academic Press; 1998 pp. 19–30. 10.1016/s0091-679x(08)61569-1 9648097

[19] Barnes D, Sato G. Methods for growth of cultured cells in serum-free medium. Anal. Biochem. 1980;102: 255–270. 6999941 10.1016/0003-2697(80)90151-7

[20] Savonniere S, Zeghari N, Miccoli L., Muller S, Maugras M, Donner M. Effects of lipid supplementation of culture media on cell growth, antibody production, membrane structure and dynamics in two hybridomas. J Biotechnol. 1996;48: 161–173. 8818281 10.1016/0168-1656(96)01392-2

[21] Sotiropoulou PA, Perez AA, Salagianni M, Baxevanis CN, Papamichail M. Characterization of the optimal culture conditions for clinical scale production of human mesenchymal stem cells. Stem Cells. 2006;24: 462–471. 10.1634/stemcells.2004-0331 16109759

[22] Benavente CA, Sierralta WD, Conget PA, Minguell JJ. Subcellular distribution and mitogenic effect of basic fibroblast growth factor in mesenchymal uncommitted stem cells. Growth Factors. 2003;21: 87–94. 14626356 10.1080/08977190310001613789

[23] Ng F, Boucher S, Koh S, Sastry KSR, Chase L, Lakshmipathy U, et al PDGF, TGFß, and FGF signaling is important for differentiation and growth of mesenchymal stem cells (MSCs): transcriptional profiling can identify markers and signaling pathways important in differentiation of MSCs into adipogenic, chondrogenic, and osteogenic lineages. Blood. 2008;112: 295–307. 10.1182/blood-2007-07-103697 18332228

[24] Mimura S, Kimura N, Hirata M, Tateyama D, Hayashida M, Umezawa A, et al Growth-factor defined culture medium for human mesenchymal stem cells. Int J Dev Biol. 2011;55: 181–187. 10.1387/ijdb.103232sm 21305471

[25] Tamama K, Kawasaki H, Wells A. Epidermal growth factor (EGF) treatment on multipotential stromal cells (MSC). Possible enhancement of therapeutic potential of MSC. J Biomed Biotechnol. 2010;2010: 795385. 10.1155/2010/795385 PMC2825653 20182548

[26] Chase LG, Lakshmipathy U, Solchaga LA, Rao MS, Vemuri MC. A novel serum-free medium for the expansion of human mesenchymal stem cells. Stem Cells Res Therapy. 2010;1: 8. 10.1186/scrt8 PMC3226302 20504289

[27] Francis GL. Albumin and mammalian cell culture: implications for biotechnology applications. Cytotechnol. 2010;62: 1–16. 10.1007/s10616-010-9263-3 PMC2860567 20373019

[28] Sakwe AM, Koumangoye R, Goodwin SJ, Ochieng J. Fetuin-A (α2HS-Glycoprotein) is a major serum adhesive protein that mediates growth signaling in breast tumor cells. J Biol Chem. 2010;85: 41827–41835. 10.1074/jbc.M110.128926 PMC3009910 20956534

[29] Croisille L, Auffray I, Katz A, Izac B, Vainchenker W, Coulombel L. Hydrocortisone differently affects the ability of murine stromal cells and human marrow-derived adherent cells to promote the differentiation of CD34++/CD38- long-term culture initiating cells. Blood. 1994;84: 4116–4124. 7527666

[30] Shipunova NN, Petinati NA, Drize NI. Effect of hydrocortisone on multipotent human mesenchymal stromal cells. Bull Exp Biol Med. 2013;155: 159–163. 23667895 10.1007/s10517-013-2102-8

[31] Kyurkchiev DS, Ivanova-Todorova E, Kyurkchiev SD. Effect of progesterone on human mesenchymal stem cells. Vitam Horm. 2011;87: 217–237. 10.1016/B978-0-12-386015-6.00040-8 22127245

[32] Choi K-M, Seo Y-K, Yoon H-H, Song K-Y, Kwon S-Y, Lee H-S, et al Effect of ascorbic acid on bone marrow-derived mesenchymal stem cell proliferation and differentiation. J Biosci Bioeng. 2008:105: 586–594. 10.1263/jbb.105.586 18640597

[33] Sato H, Takahashi M, Ise H, Yamada A, Hirose S, Tagawa Y, et al Collagen synthesis is required for ascorbic acid-enhanced differentiation of mouse embryonic stem cells into cardiomyocytes. Biochem Biophys Res Commun. 2006;342: 107–112. 10.1016/j.bbrc.2006.01.116 16480687

[34] Igarashi I, Kashiwagi K. Modulation of cellular function by polyamines. Int J Biochem Cell Biol. 2010;42: 39–51. 10.1016/j.biocel.2009.07.009 19643201

[35] Zhang D, Zhao T, Ang HS, Chong P, Saiki R, Igarashi K, et al AMD1 is essential for ESC self-renewal and is translationally down-regulated on differentiation to neural precursor cells. Genes Dev. 2012;26: 461–473. 10.1101/gad.182998.111 22391449 PMC3305984

[36] Fraser JK, Zhu M, Wulur I, Alfonso Z. Adipose-derived stem cells In: Prockop DJ, Phinney DG, Bunnell BA, editors. Methods in Mol. Biol. Mesenchymal stem cells: methods and protocols. Totowa, NJ: Humana Press; 2008 pp. 59–68. 10.1007/978-1-60327-169-1_4 18370083

[37] Screven R., Kenyon E., Myers M.J., Yancy H.F., Skasko M., Boxer, et al Immunophenotype and gene expression profile of mesenchymal stem cells derived from canine adipose tissue and bone marrow. Vet Immunol Immunopathol. 2014;161: 21–31. 10.1016/j.vetimm.2014.06.002 25026887

[38] Colleoni S, Bottani E, Tessaro I, Mari G, Merlo B, Romagnoli N, et al Isolation, growth and differentiation of equine mesenchymal stem cells: effect of donor, source, amount of tissue and supplementation with basis fibroblast growth factor. Vet Res Commun. 2009;33: 811–821. 10.1007/s11259-009-9229-0 19472068

[39] Wodewotzky TI, Lima-Neto JF, Pereira-Junior OC, Sudano MJ, Lima SA, Bersano PR, et al In vitro cultivation of canine multipotent mesenchymal stromal cells on collagen membranes treated with hyaluronic acid for cell therapy and tissue regeneration. Braz J Med Biol Res. 2012;45: 1157–1162. 10.1590/S0100-879X2012007500149 22983182 PMC3854207

[40] Nakano R, Edamura K, Nakayama T, Teshima K, Asano K, Narita T, et al Differentiation of canine bone marrow stromal cells into voltage- and glutamate-responsive neuron-like cells by basic fibroblast growth factor. J Vet Med Sci. 2015;77: 27–35. 10.1292/jvms.14-0284 25284120 PMC4349535

[41] Jung S, Panchalingam KM, Rosenberg L, Behie LA. Ex vivo expansion of human mesenchymal stem cells in defined serum-free media. Stem Cells Int. 2012;2012: 123030. 10.1155/2012/123030 PMC3356989 22645619

[42] Pittenger MF, Mackay AM, Beck SC, Jaiswal RK, Douglas R, Mosca JD, et al Multilineage potential of adult human mesenchymal stem cells. Science. 1999;284: 143–147. 10102814 10.1126/science.284.5411.143

[43] Liu Z, Screven R, Myers M, Devireddy LR. Characterization of canine adipose-derived mesenchymal stem/stromal cells in serum-free medium. Tissue Eng Part C. 2018;24: 399–411. 10.1089/ten.TEC.2017.0409 29770732

