# Supplementary figures and images for: A serum-free medium formulation efficiently supports isolation and propagation of canine adipose-derived mesenchymal stem/stromal cells
Source: PLoS One. 2019 Feb 27;14(2):e0210250. doi: 10.1371/journal.pone.0210250 (PMC6392232; doi:10.1371/journal.pone.0210250)

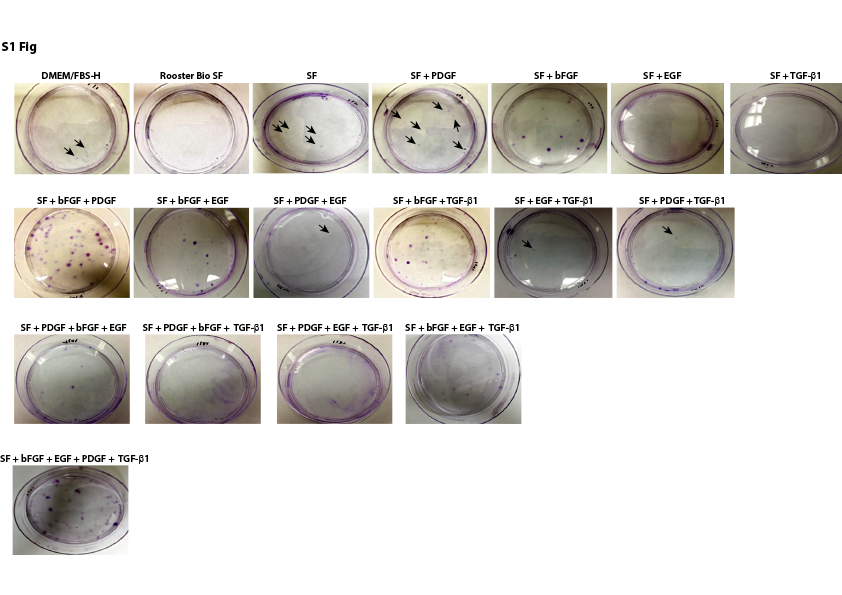

Supplement: S1 Fig — Images of canine Ad-MSC colonies cultured in serum-free medium with or without growth factors or in serum-containing medium. Small colonies are indicated by arrows. Note: Colonies in TGF-ß1 supplemented serum-free medium are very faint. (TIF) [file pone.0210250.s001.tif]
